# Oral and oropharyngeal mucosal lesions: clinical-epidemiological study of patients attended at a reference center for infectious diseases

**DOI:** 10.1016/j.bjorl.2024.101396

**Published:** 2024-02-01

**Authors:** Clarissa Souza Mota Reis, João Gustavo Corrêa Reis, Fátima Conceição-Silva, Cláudia Maria Valete

**Affiliations:** a Fundação Oswaldo Cruz (FIOCRUZ), Instituto Nacional de Infectologia Evandro Chagas (INI), Rio de Janeiro, RJ, Brazil; b Fundação Oswaldo Cruz (FIOCRUZ), Instituto Oswaldo Cruz (IOC), Laboratório de Imunoparasitologia, Rio de Janeiro, RJ, Brazil; c Hospital Federal de Bonsucesso, Departamento de Broncoesofagolaringologia e Cirurgia de Cabeça e Pescoço, Rio de Janeiro, RJ, Brazil; d Universidade Federal do Rio de Janeiro, Faculdade de Medicina, Departamento de Otorrinolaringologia e Oftalmologia, Rio de Janeiro, RJ, Brazil

**Keywords:** Oral manifestations, Oropharynx, Mouth diseases, Differential diagnosis, Infectious disease medicine

## Abstract

**Objective:**

To determine the prevalence, epidemiological profile, and clinical characteristics of Oral or Oropharyngeal Mucosal Lesions (OOPML) in patients attended at the Otorhinolaryngology Service of the Evandro Chagas National Institute of Infectious Diseases (INI-FIOCRUZ) from 2005 to 2017.

**Methods:**

Statistical analysis of descriptive data from medical records (gender, age, education level, skin color, origin, smoking, alcoholism, HIV co-infection, time of disease evolution, first symptom, and OOPML location) was performed.

**Results:**

Of 7551 patients attended at the service, 620 (8.2%) were included in the study. OOPML were classified into developmental anomalies (n = 3), infectious diseases (non-granulomatous n = 220; granulomatous n = 155), autoimmune diseases (n = 24), neoplasms (benign n = 13; malignant, n = 103), and unclassified epithelial/soft tissue diseases (n = 102). OOPML of infectious diseases (60.5%) and neoplasms (18.7%) were the most frequent. The predominant demographics of patients with OOPML were: males (63.5%), white (53.5%), and those in the fifth to sixth decades of life (43.3%). Local pain (18.1%) and odynophagia (15%) were the most reported first symptoms, and the most frequent OOPML sites were the palatine tonsil (28.5%), hard palate (22.7%), and tongue (20.3%). The median evolution time was three months.

**Conclusions:**

Infectious OOPML were the most frequent, as expected in a reference center for infectious diseases, and thus, they are likely to be less frequent in general care and/or dental services. Underreporting of OOPML is possible, as oral/oropharyngeal examination is often not included in the routine medical examination. Oral cavity/oropharynx examination should be performed by specialists, such as dentists and otorhinolaryngologists, who have the expertise in identifying OOPML, even in incipient/asymptomatic cases. Given the numerous diseases in which OOPML can present, diagnosis could be facilitated by multidisciplinary teams, potentially enabling the early treatment of diseases, and thus, reduce morbidity and improve prognosis. The use of standardized medical records for oral/oropharyngeal systematic examination could provide relevant tools for differential diagnoses and information for new clinical-epidemiological studies.

**Level of evidence:**

Level 3.

## Highlights

• Oral mucosal lesions of infectious diseases and neoplasms were the most frequent.
• Clinical-epidemiological characteristics of oral manifestations are often similar.
• Systematic oral and oropharyngeal examination is essential for differential diagnosis.
• Multidisciplinary teams in medical routine can improve early diagnosis.
• Standardized medical records can provide tools for differential diagnosis.

## Introduction

The oral cavity plays an important role in the physiology of the human organism, emphasized by the popular saying, ‘health comes first, and it enters through the mouth’. The anatomical and functional continuity between the oral cavity and oropharynx highlights the need to understand lesions of these anatomical areas. Oral or Oropharyngeal Mucosal Lesions (OOPML) include any mucosal alteration of the oral cavity/oropharynx, which may result from developmental disturbances, infections, allergic or inflammatory processes, neoplasms, or other histomorphological alterations of the epithelium and soft tissues. OOPML can be caused by primary diseases of the oral cavity/oropharynx or be clinical expressions of other organ or systemic diseases (e.g., autoimmune, infectious, or neoplastic). Therefore, OOPML may be the primary, most significant, or unique signs of diseases, leading to direct or indirect consequences on the individual's health.^1^, ^2^, ^3^, ^4^ According to the World Health Organization (WHO), oral diseases affect 3.5 billion people worldwide and the number of cases is increasing globally.^5^ Thus, a complete, systematic evaluation of the oral cavity/oropharynx is essential for the diagnosis and follow-up of primary diseases of the Upper Aerodigestive (UAD) tract or of other origins.

The data derived from the study of the clinical-epidemiological characteristics of OOPML can assist health professionals in the clinical and laboratory evaluation of patients. Our objective was to determine OOPML prevalence and anatomical location, and to describe the epidemiological profile of the patients, in addition to the first symptom presented, the diagnostic conclusion, and the time of disease evolution.

## Methods

A retrospective cross-sectional study of 7551 medical records was performed, and patients with OOPML attended at the Otorhinolaryngology Service of the Evandro Chagas National Institute of Infectious Diseases (INI-FIOCRUZ) from January 2005 to December 2017 were included in the study. Clinical and epidemiological data were collected and stored in a database for statistical analysis. This study was approved by the Research Ethics Committee of INI-FIOCRUZ under protocol number 759873179.0000.5262.

The criteria used for diagnostic confirmation were the presence of OOPML associated with the patient's medical history, clinical characteristics of the lesion, serological tests, direct or histopathological examinations or the culture of specimens obtained from the oral cavity, oropharynx, or other anatomical sites with concomitant manifestations, or clinical/radiological suspicion associated with OOPML remission after specific treatment (Table 1).

**Table 1:** Diagnoses and the diagnostic methods of patients with oral or oropharyngeal lesions among the 7551 patients attended at the Otorhinolaryngology Service of the Evandro Chagas National Institute of Infectious Diseases (INI-FIOCRUZ), from 2005 to 2017.

Disease	Diagnosis
Leukoedema	Clinical
Lymphoepithelial cyst	Clinical
Necrotizing ulcerative gingivitis	Clinical
Peritonsillar abscess	Clinical
Herpes	Clinical, associated or not with serology
Syphilis	Clinical, associated with positivity of non-treponemal (VDRL) and treponemal serological tests (FTA-ABS and TPHA)
Recurrent tonsillitis	Clinical (5 episodes per year for 2 consecutive years or 3 episodes per year for 3 consecutive years)
Acute tonsillitis and pharyngitis	Clinical
Candidiasis	Clinical, with or without culture
Leprosy	Clinical and histopathology
Histoplasmosis	Clinical and histopathology
Sporotrichosis	Clinical and direct examination, culture, or histopathology
Tuberculosis	Clinical and direct examination or culture (tissue specimen or sputum), histopathology, chest X-Ray, resolution with treatment
American tegumentary leishmaniasis	Clinical, associated with serology, Montenegro´s skin test, direct examination, culture, PCR, or histopathology
Paracoccidioidomycosis	Clinical, associated with serology, direct examination, culture, or histopathology
Behçet's disease	Clinical (recurrent oral ulcers associated with two of the following manifestations: recurrent genital ulcers, eye lesions, or skin lesions)
Mucous membrane pemphigoid	Clinical and histopathology
Pemphigus vulgaris	Clinical and histopathology
Lichen planus	Clinical, with or without histopathology
Benign neoplasm	Clinical and histopathology
Malignant neoplasm	Clinical and histopathology, with or without immunohistochemistry. Exception: leukemia - diagnosis by peripheral blood study
Mucocele and ranula	Clinical, with or without histopathology
Hypertrophy of lingual and palatine tonsils	Clinical
Pyogenic granuloma	Clinical and histopathology
Benign migratory glossitis	Clinical
Leukoplakia	Clinical
Fibroma	Clinical and histopathology
Nonspecific ulcerated lesion	Clinical, lesions that resolved spontaneously or lesions without specific treatment (e.g., traumatic ulcers and recurrent aphthous stomatitis)

OOPML presented by the patients were classified into developmental disturbances, Non-Granulomatous (NGID) and Granulomatous Infectious Diseases (GID), autoimmune diseases, neoplasms (benign and malignant), and epithelial and soft tissue diseases Not Classified in Other Categories (NCOC). The inclusion of OOPML in this last category was based on the classification criteria of Neville et al. (2016).^7^ Syphilis was classified as a NGID, owing to its nonspecific histopathological pattern in the primary and secondary stages. Nonspecific ulcerated lesions were classified as those that resolved spontaneously or without specific treatment. The prevalence of dental caries and periodontal disease was not evaluated. The clinical-epidemiological variables used are given in Supplemental Table S1.

Two classifications regarding OOPML location were used: general and oral cavity/oropharyngeal subsites. The definition of subsites followed the anatomical division proposed by the TNM classification of malignant tumors,^8^ with the following modifications: tonsillar pocket and tonsil were considered as “tonsil”; uvula was considered as “soft palate”; and upper/lower lip and labial commissure were considered as “oral only”.

The Statistical Package for Social Science (SPSS) for Windows, version 16.0 (SPSS Inc., Chicago, IL, USA), was used for data analysis. The simple frequencies of categorical variables were determined, as well as the summary measures (mean ± Standard Deviation [SD], median, Interquartile Range [IQR], and minimum and maximum) of continuous variables.

## Results

**Fig. 1:**
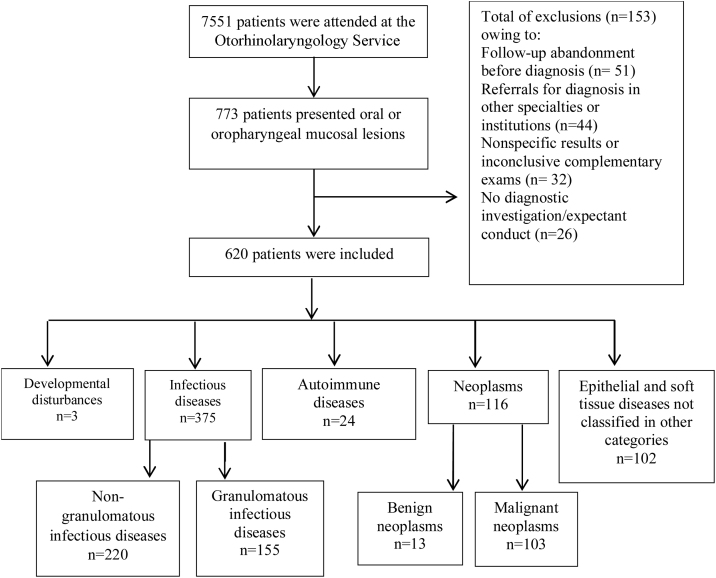
Flowchart of the selection of patients with oral or oropharyngeal lesions among the 7551 patients attended at the Otorhinolaryngology Service of the Evandro Chagas National Institute of Infectious Diseases (INI-FIOCRUZ), from 2005 to 2017.

A total of 7551 medical records were reviewed and 773 (10.2%) patients had OOPML (Fig. 1).

Patients included in the study ranged from 1 to 92 years of age (median = 47, IQR = 31–57). The mean age and range (in years) of patients in each disease group were: developmental disturbances, 51 ± 6 and 45–57; NGID, 36.6 ± 16.7 and 1–77; GID, 51.1 ± 12.9 and 15–80; autoimmune diseases, 53.6 ± 16.5 and 21–84; benign neoplasms, 41.8 ± 10 and 21–55; malignant neoplasms, 57 ± 15.3 and 20–92; and NCOC, 39 ± 19.2 and 3–78. The distribution of age, by age group, is available in Supplemental Fig. S1 and Supplemental Table S2. Epidemiological characteristics of patients with OOPML are given in Table 2.

**Table 2:** Epidemiological characteristics of patients with oral or oropharyngeal mucosal lesions attended at the Otorhinolaryngology Service of the Evandro Chagas National Institute of Infectious Diseases (INI-FIOCRUZ), from 2005 to 2017.

		Developmental disturbances		Non-granulomatous infectious diseases		Granulomatous infectious diseases		Autoimmune diseases		Benign neoplasms		Malignant neoplasms		NCOC diseases^a^		Total	
		n	%	n	%	n	%	n	%	n	%	n	%	n	%	n	%
Gender (n^b^ = 620)	Female	2	66.7	123	55.9	24	15.5	15	62.5	4	30.8	19	18.4	39	38.2	226	36.5
	Male	1	33.3	97	44.1	131	84.5	9	37.5	9	69.2	84	81.6	63	61.8	394	63.5
Skin color (n = 477)	White	0	0	99	60.0	66	43.4	12	75.0	5	45.5	33	50.8	40	61.5	255	53.5
	Feoderm	0	0	46	27.9	63	41.4	4	25.0	6	54.5	21	32.3	17	26.2	157	32.9
	Black	3	100	20	12.1	23	15.2	0	0	0	0	11	16.9	8	12.3	165	13.6
Education level (n = 547)	Elementary school^c^	2	66.7	47	24.1	88	58.7	8	40.0	1	9.1	42	50.0	27	32.1	215	39.3
	Middle school	0	0	34	17.4	37	24.7	4	20.0	1	9.1	17	20.2	17	20.2	110	20.1
	High school-Graduate school^d^	1	33.3	114	58.5	25	16.6	8	40.0	9	81.8	25	29.8	40	47.6	222	40.6
Origin (n = 620)	Rio de Janeiro city^e^	2	66.7	205	93.2	109	70.3	20	83.3	12	92.3	88	85.4	98	96.1	534	86.1
	Rio de Janeiro state	0	0	13	5.9	44	28.4	0	0	1	7.7	11	10.7	4	3.9	73	11.8
	Other states^f^	1	33.3	2	0.9	2	1.3	4	16.7	0	0	4	3.9	0	0	13	2.1
Smoking (n = 211)	Smoking	0	0	13	76.5	57	62.6	1	12.5	3	100	50	63.3	8	61.5	232	62.6
Alcohol use (n = 128)	Alcohol use	1	100	3	27.3	24	38.1	1	25.0	1	100	16	36.4	0	0	46	35.9

a NCOC diseases - epithelial and soft tissue diseases not classified in other categories.

b Number of patients with available information.

c Range from illiterate up to last year of elementary school.

d High school, associate degree, undergraduate degree, and graduate school.

e Rio de Janeiro city and metropolitan region.

f Other states of Brazil.

For GID cases, given the potential link to rural areas, information regarding residence/labor activity in urban/rural areas was collated. Data were available for 123 (79.4%) patients, comprising 86 (69.9%) from urban areas and 37 (30.1%) from rural areas (Supplemental Table S3).

Simultaneous involvement of the oral cavity/oropharynx was uncommon in the patients included in this study (12.6%) (Table 3).

**Table 3:** General location of oral or oropharyngeal mucosal lesions of patients attended at the Otorhinolaryngology Service of the Evandro Chagas National Institute of Infectious Diseases (INI-FIOCRUZ), from 2005 to 2017.

	Developmental disturbances (n = 3)		Non-granulomatous infectious diseases (n = 220)		Granulomatous infectious diseases (n = 155)		Autoimmune Diseases (n = 24)		Benign neoplasms (n = 13)		Malignant neoplasms (n = 103)		NCOC diseases^a^ (n = 102)		Total (n = 620)	
	n	%	n	%	n	%	n	%	n	%	n	%	n	%	n	%
Oral only	1	33.3	84	38.2	68	43.9	18	75	10	76.9	41	39.8	68	66.7	290	46.8
Oropharyngeal only	2	66.7	126	57.3	41	26.5	0	0	3	23.1	49	47.6	31	30.4	252	40.6
Oral and oropharyngeal	0	0	10	4.5	46	29.7	6	25	0	0	13	12.6	3	2.9	78	12.6

a NCOC diseases - epithelial and soft tissue diseases not classified in other categories.

The most affected oral/oropharyngeal subsites, in descending order, were the palatine tonsil, hard palate, tongue, and soft palate (Table 4).

**Table 4:** Subsites of oral or oropharyngeal mucosal lesions of patients attended at the Otorhinolaryngology Service of the Evandro Chagas National Institute of Infectious Diseases (INI-FIOCRUZ), from 2005 to 2017.

Subsites^a^	Developmental disturbances (n = 3)		Non-granulomatous infectious diseases (n = 220)		Granulomatous infectious diseases (n = 155)		Autoimmune diseases (n = 24)		Benign neoplasms (n = 13)		Malignant neoplasms (n = 103)		NCOC diseases^b^ (n = 102)		Total (n = 620)	
	n	%	n	%	n	%	n	%	n	%	n	%	n	%	n	%
Upper lip	0	0	6	2.7	17	10.9	3	12.5	1	7.7	2	1.9	3	2.9	32	5.2
Cutaneous extension	0	0	0	0	11	7	0	0	0	0	0	0	0	0	11	1.8
Lower lip	0	0	8	3.6	14	9	4	16.6	2	15.4	4	3.9	14	13.7	46	7.5
Labial commissure	0	0	4	1.8	5	3.2	1	4.2	0	0	0	0	0	0	10	1.6
Tongue	0	0	28	12.7	18	11.6	10	41.7	1	7.7	20	19.4	32	31.4	109	17.6
Floor of mouth	0	0	1	0.4	5	3.2	0	0	0	0	3	2.9	3	2.9	12	1.9
Upper gum	0	0	8	3.6	23	14.8	5	20.8	0	0	4	3.9	5	4.9	45	7.25
Retromolar trigone	0	0	1	0.4	3	1.9	1	4.2	0	0	4	3.9	1	1	10	1.6
Buccal mucosa	1	33.3	9	4	10	6.4	13	54.2	5	38.5	5	4.85	5	4.9	48	7.7
Lower gum	0	0	8	3.6	21	13.5	5	20.8	0	0	2	1.9	6	6.9	42	6.8
Hard palate	0	0	46	20.9	46	29.7	5	20.8	1	7.7	17	16.5	7	6.9	122	19.7
Soft palate	0	0	11	5	59	38	4	16.6	3	23	27	26.2	3	2.9	107	17.2
Lingual tonsils	0	0	0	0	3	1.9	0	0	1	7.7	10	9.7	4	3.9	18	2.9
Anterior tonsillar pillar	1	33.3	3	1.4	26	16.7	3	12.5	0	0	21	20.4	5	4.9	59	9.5
Posterior tonsillar pillar	1	33.3	2	0.9	14	9	1	4.2	0	0	8	7.8	1	1	27	4.3
Palatine tonsils	0	0	81	36.8	22	14.1	1	4.2	0	0	27	26.2	22	21.6	153	24.7
Posterior pharyngeal wall	0	0	6	2.7	25	16.1	0	0	0	0	4	3.9	1	1	36	5.8

a May be more than 1 subsite per patient.

b NCOC diseases - epithelial and soft tissue diseases not classified in other categories.

Data on the first mucosal sign/symptom presented by patients with OOPML were available for 286 (46.1%) patients, and local pain and odynophagia were the most common. Information on HIV co-infection was recorded for 203 (32.7%) patients and more than half of these were HIV-positive (Supplemental Tables S4 and S5).

**Fig. 2:**
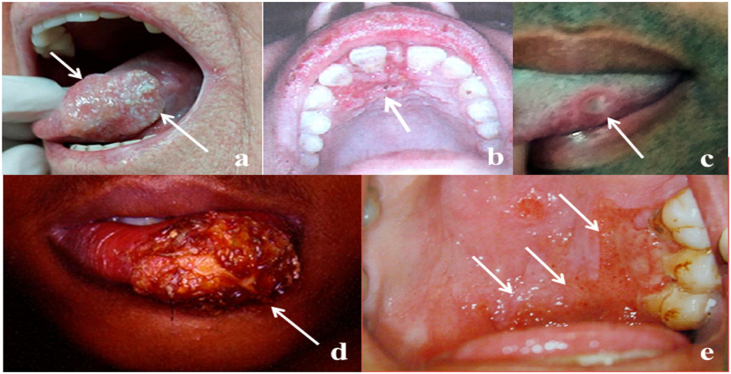
Representative images of OOPML of patients attended at the Otorhinolaryngology Service of the Evandro Chagas National Institute of Infectious Diseases (INI-FIOCRUZ), from 2005 to 2017: (a) Squamous cell carcinoma (arrows); (b) tuberculosis (arrow); (c) syphilis (arrow); (d) American tegumentary leishmaniasis (arrow); and (e) paracoccidioidomycosis (arrows).

The frequency of OOPML for all included patients is shown in Table 5. The time of disease evolution was reported for 273 (44%) patients, with the median time being 3-months (IQR = 1–6). The time range for each group was as follows: NGID, 0.10–4 months (median = 0.25, IQR = 0.15–1); GID, 0.16–120 months (median = 6, IQR = 3–12); autoimmune diseases, 0.5–8 months (median = 3, IQR = 2–7); benign neoplasms, 1–240 months (median = 0.25, IQR = 1.5–138); malignant neoplasms, 0.75–60 months (median = 3, IQR = 2–6); and NCOC, 0.25–36 months (median = 2, IQR = 0.31–4). The time information was available for only one patient (4-months) of the developmental disturbances group, who had a lymphoepithelial cyst. Patients with GID, malignant neoplasms, and autoimmune diseases were seen more frequently from the third month after the appearance of the first mucosal sign/symptom. Images of some OOPML are presented in Fig. 2.

**Table 5:** Frequency of oral or oropharyngeal mucosal lesions of patients attended at the Otorhinolaryngology Service of the Evandro Chagas National Institute of Infectious Diseases (INI-FIOCRUZ), from 2005 to 2017.

Classification	Diseases	n	%
Developmental disturbances	Leukoedema	1	0.2
	Lymphoepithelial cyst	2	0.3
Non-granulomatous infectious diseases	Necrotizing ulcerative gingivitis	2	0.3
	Peritonsillar abscess	6	1.0
	Herpes	12	1.9
	Syphilis	18	2.9
	Recurrent tonsillitis	25	4.0
	Acute pharyngitis	39	6.3
	Acute tonsillitis	48	7.7
	Candidiasis	70	11.3
Granulomatous infectious diseases	Leprosy	2	0.3
	Histoplasmosis	5	0.8
	Sporotrichosis	5	0.8
	Tuberculosis	13	2.1
	American tegumentary leishmaniasis	55	8.9
	Paracoccidioidomycosis	75	12.1
Autoimmune diseases	Behcet's disease	2	0.3
	Mucous membrane pemphigoid	4	0.6
	Pemphigus vulgaris	8	1.3
	Lichen planus	10	1.6
Benign neoplasms	Pleomorphic adenoma	1	0.2
	Squamous papilloma	12	1.9
Malignant neoplasms	Leukemia	1	0.2
	Neoplasm without definition of histological pattern	7	1.1
	Natural killer/T-cell lymphoma	1	0.2
	Lymphoma	2	0.3
	Non-Hodgkin lymphoma	2	0.3
	Kaposi sarcoma	10	1.6
	Adenoid cystic carcinoma	1	0.2
	Carcinoma in situ	2	0.3
	Squamous cell carcinoma	77	12.4
Epithelial and soft tissue diseases not classified in other categories	Ranula	1	0.2
	Mucocele	3	0.5
	Lingual tonsillar hypertrophy	3	0.5
	Pyogenic granuloma	4	0.6
	Benign migratory glossitis	6	1.0
	Leukoplakia	12	1.9
	Fibroma	14	2.3
	Palatine tonsillar hypertrophy	20	3.2
	Nonspecific ulcerated lesion	39	6.3
	Total	620	100.0

## Discussion

OOPML were observed in 10.2% of all patients evaluated during the study period. Most of the identified cases were infectious diseases (Mainly Paracoccidioidomycosis [PCM], candidiasis, and American Tegumentary Leishmaniasis [ATL]), followed by malignant neoplasms. The data collected in this study come from a reference center for infectious diseases, which could explain the high percentage of infectious disease related OOPML. This did not, however, prevent the diagnosis of a variety of non-infectious diseases, which in itself demonstrates the difficulty of OOPML differential diagnosis, since most of these patients were referred to our center as a suspected infectious disease case. In other epidemiological surveys, oral lesions of non-odontogenic/non-periodontal infectious diseases range from 0.8% to 23.2%, mostly restricted to herpes, PCM, and candidiasis, with the latter being the most frequent.^9^, ^10^, ^11^, ^12^, ^13^

The prevalence of oral lesions is primarily determined through population-based studies,^14^, ^15^, ^16^, ^17^, ^18^ or studies carried out in dental centers^19^, ^20^, ^21^ or from oral pathology laboratories.^22^, ^23^, ^24^ However, as no standardization in OOPML classification exists, with studies classifying OOPML by a lesion group (e.g., non-neoplastic lesions)^19^ or by a specific disease (e.g., oral lesions in syphilis),^25^ the reported OOPML frequencies are directly influenced.^26^, ^27^, ^28^ Furthermore, the lack of systematic and standardized inclusion of oral cavity/oropharynx examination in the routine medical examination generates gaps in medical records,^29^, ^30^ and epidemiological surveys on oral health only provide information on diseases related to dental elements (e.g., caries, edentulism).^5^, ^31^ Despite the limitations related to retrospective studies, the present study from an otorhinolaryngology service provides OOPML prevalence data for numerous diseases that were diagnosed.

The prevalence of OOPML reported in studies can be influenced by the country or geographic region in which the study is conducted, the socioeconomic level of patients, and the methodologies used, which may explain the large variation observed among publications (4.9%–64.5%).^14^, ^15^, ^16^, ^17^, ^18^ Our study demonstrated that 10.2% of the total number of patients seen at the Otorhinolaryngology Service of INI-FIOCRUZ had OOPML, similar to the prevalence observed in population-based studies.^16^, ^18^

Our sample predominantly comprised white, male individuals, in the fifth and sixth decades of life. Age group and skin color can vary depending on the study, as some studies on OOPML report a similar distribution,^13^, ^16^, ^21^, ^32^ while others report a female majority.^6^, ^9^, ^14^, ^16^^,^^32^ The male predominance in the present study may be related to the higher frequency of GID and Squamous Cell Carcinoma (SCC), diseases which are more common in men;^33^, ^34^, ^35^ in contrast, benign lesions, mainly inflammatory fibrous hyperplasia, occur more frequently in women.^6^, ^9^, ^13^ Most of our patients had low education levels similar to that observed by Souza et al. (2017). Lower levels of education have been associated with infectious and neoplastic diseases of the UAD tract, which were very frequent in our sample.^36^, ^37^, ^38^

Smoking and alcohol use are generally associated with an increased OOPML incidence in GID and malignant neoplasms.^17^, ^18^, ^38^, ^39^ However, data on these factors were only available in 34% and 20.6% of patients, respectively. Although studies use different concepts of smoking and alcohol use, our study only considered the reference to smoking or alcohol consumption in the medical records in the data collection and, from this, we were able to observe similar frequencies of smokers and drinkers as in other studies.^13^, ^21^

Local pain and odynophagia were the first symptoms most reported by patients, whereas in the study by Santos et al. (2013), most patients were asymptomatic.^21^ This difference can be attributed to the most frequent type of lesion found. Santos et al. (2013) reported OOPML of inflammatory fibrous hyperplasia as the most frequent, a disease which is usually asymptomatic, whereas in our study, OOPML of SCC, autoimmune diseases, acute tonsillitis, and pharyngitis, which are usually associated with local pain and/or odynophagia, were more prevalent.^40^, ^41^, ^42^

The most frequently affected oral/oropharyngeal subsites were, in descending order, the palatine tonsil, hard palate, tongue, and soft palate, probably influenced by the frequency of acute tonsillitis/pharyngitis, autoimmune diseases, GID, and SCC. Likewise, the OOPML locations in other epidemiological surveys varied according to the diseases observed.^13^, ^14^, ^20^, ^21^^,^^32^ It is worth noting that the anatomical division of the oral cavity/oropharynx between studies is not standardized.^6^, ^9^, ^13^ As an example, the soft palate, considered as an oral cavity subsite by some authors,^6^, ^13^, ^21^ was considered as oropharynx in the present study based on TNM anatomical division criteria.^8^

The median time of disease evolution indicated that patients take approximately three months until the first medical consultation, similar to that reported by Santos et al.^21^ This extended waiting time could affect early diagnosis, which is important in reducing sequelae.^39^, ^43^ A longer evolution time was observed for patients with GID and neoplasms, which are chronic diseases, often with an insidious and initially oligosymptomatic evolution. As such, patients may delay seeking medical care, in addition, accessing medical resources and laboratory tests for their diagnoses may be hindered. Conversely, NGID generally have more intense and rapidly evolving symptoms, encouraging patients to seek medical care earlier.

Lymphoepithelial cyst, leukoedema, ranula, mucocele, pyogenic granuloma, benign migratory glossitis, fibroma, and leukoplakia were observed at lower frequencies than in other OOPML epidemiological surveys, probably because patients with these OOPML are usually treated in dental, rather than otorhinolaryngology, services.^6^, ^9^, ^19^, ^20^^,^^27^, ^32^

The frequency of autoimmune disease cases observed in the present study corroborates that of Carvalho et al. (2011). In both studies, the immunologically mediated dermatological diseases with OOPML were diagnosed as Lichen Planus (LP), pemphigus vulgaris, and mucous membrane pemphigoid.^44^ LP OOPML were the most frequent, as in other studies.^14^, ^16^, ^17^, ^45^ However, these lesions can still be considered rare, since the global prevalence is ∼1%.^46^

Almost all the benign neoplasm-related OOPML were diagnosed as squamous papilloma. The presence of this OOPML may be related to HIV infection, since most of these patients were carriers of the virus, a population at greater risk of HPV infection.^47^ The frequency of squamous papilloma was similar to that observed in other studies, although the rate of immunosuppression was not reported.^13^, ^17^, ^32^

Regarding malignant neoplasms, the occurrence of SCC was equal to or greater than that reported in other OOPML surveys.^9^, ^20^, ^32^ The high frequency of this disease may be a consequence of the similarity between the clinical and epidemiological characteristics of SCC OOPML with those of GID, which justifies the referral of these patients to our center. This emphasizes the importance of biopsies for diagnosis, to rule out concomitant lesions of other etiologies, and to investigate the association of SCC with HPV. HPV is an important risk factor for SCC, especially for oropharyngeal cases, and an increase in HPV-positive cancers has been observed in Brazilian cohorts.^48^, ^49^

As in other studies, the total OOPML frequency in the different lymphomas was <1%, confirming its rarity.^50^, ^51^ We observed a higher frequency of OOPML from sarcomas than that observed in neoplasms of hematological origin, unlike what was reported by Allon et al.^40^ HIV co-infection may have influenced this difference, since the literature demonstrates that OOPML can be observed in up to half of patients with Kaposi sarcoma and AIDS.^3^, ^52^, ^53^

We observed nonspecific ulcerated lesions at frequencies similar to those reported by other studies for traumatic ulcerations and recurrent aphthous stomatitis.^13^, ^14^, ^17^, ^18^^,^^21^, ^32^, ^54^ Ulcerated lesions may be underdiagnosed because their course is short and self-limited, meaning that most patients do not seek medical care.

Candidiasis and PCM were the most frequently observed diagnoses in cases of NGID- and GID-related OOPML, respectively. We found a higher frequency of candidiasis than that reported in other OOPML surveys with no defined age group. This possibly occurred owing to the higher frequency of HIV co-infection in our sample.^9^, ^13^, ^45^ However, the observed frequency of this fungal infection was lower than that shown in surveys conducted in older adults.^11^, ^45^ Most patients in our sample were in their fifth and sixth decades of life, and the use of dental prostheses, which has been linked to these infections, was possibly lower.

Acute tonsillitis and pharyngitis were also frequent in the NGID group, and predominantly occurred in patients in their second and third decades of life, in contrast to that observed in other studies, which have reported a higher prevalence in children and adolescents.^10^, ^42^, ^55^ This difference in age groups can be attributed to the fact that our service mainly meets the demand of adult patients.

OOPML of syphilis were uncommon in our sample, despite the increase in the number of syphilis cases in recent years.^56^, ^57^ The low prevalence in the current study may be owing to the fact that patients with clinical suspicion of this disease are routinely treated at Sexually Transmitted Disease/AIDS Outpatient Clinics, and, as OOPML improvement typically occurs with the beginning of treatment, patients do not seek evaluation at other services. No cases of oral syphilis lesions have been reported in any other OOPML survey.^16^, ^20^, ^58^ Despite syphilis being a notifiable disease in Brazil, the OOPML prevalence of this disease is likely underreported, since the clinical form of the Notifiable Diseases Information System does not include the registration of OOPML.^56^

The low frequency of herpes was similar to that observed in other OOPML surveys.^12^, ^13^, ^17^, ^59^ As herpes OOPML are usually recurrent and immunocompetent patients are already familiar with the self-limited evolution,^60^ they do not typically seek medical or dental care.

The oral cavity/oropharynx are commonly affected in PCM.^61^, ^62^ The PCM OOPML frequency in our study was proportionally higher than that of other studies when considering the duration of the studies,^51^, ^62^ including that observed in a study carried out in a region with high PCM prevalence.^63^ This higher prevalence is likely owing to the fact that our service is a reference center for infectious diseases and conducts the systematic otorhinolaryngological examination of patients referred by other services. For the same reason, ATL was the second most frequent diagnosis in GID-related OOPML cases. In this disease, the oral cavity/oropharynx are the second most affected anatomical sites in the head and neck.^43^, ^64^ Underreporting of these OOPML may also occur as a result of the lack of oral/oropharyngeal examination in the medical routine.

The frequency of tuberculosis OOPML was slightly higher than that observed in the literature,^65^, ^66^ which is likely related to the systematic oral/oropharyngeal examination performed at our otorhinolaryngology service. Overall, the prevalence of tuberculosis OOPML is difficult to estimate owing to the low frequency,^65^, ^66^ in addition to the lack of data in official reports, which generally only report the incidence of extrapulmonary forms of the disease.^67^, ^68^

Studies that provide the prevalence of histoplasmosis OOPML report the percentage of these lesions in patients with the disease and not in the general population.^69^, ^70^ Despite sporotrichosis being an endemic disease in the state of Rio de Janeiro,^71^ the frequency of OOPML was low in our study, confirming the rarity of lesions in this disease.^72^, ^73^ In addition to OOPML in leprosy being rare,^74^ the low frequency in the present study may be related to the fact that most patients are routinely treated at specific leprosy reference centers.^75^

## Conclusions

Diseases that affect the oral cavity/oropharynx are the subject of study in several areas of health sciences, such as dentistry, otorhinolaryngology, and dermatology. For this reason, lesions in these anatomical areas are often evaluated in a fragmented way. Studies on the general prevalence of OOPML are scarce and surveys are often carried out for specific disease groups or by dental centers. Like dentists, otolaryngologists may be the first professionals to identify OOPML. Therefore, the organization of multidisciplinary teams that include otolaryngologists for routine UAD tract examinations, even in asymptomatic cases, could facilitate the early diagnosis and treatment of many diseases, thus reducing morbidity and improving the prognosis, as in many cases, patients only show symptoms when in a more advanced stage. In addition, the use of standardized medical records for systematic examination of the oral cavity/oropharynx can provide tools for differential diagnosis and relevant information for new clinical-epidemiological studies.

## Funding

This research was funded by FIOCRUZ (grant number PAEF-IOC 008-F10—22-2-49) and FAPERJ (grant number APQ1 FAPERJ-E-26/21 -707/2021). C.S.M. Reis is a PhD student in Clinical Research in Infectious Diseases at INI-FIOCRUZ. The funding sources had no involvement in the collection, analysis and interpretation of data; in the writing of the report; and in the decision to submit the article for publication.

## Conflicts of interest

The authors declare no have conflicts of interest.
