# Supplementary material for: Oral and oropharyngeal mucosal lesions: clinical-epidemiological study of patients attended at a reference center for infectious diseases
Source: Braz J Otorhinolaryngol. 2024 Feb 1;90(3):101396. doi: 10.1016/j.bjorl.2024.101396 (PMC10877199; doi:10.1016/j.bjorl.2024.101396)
Supplement: Supplementary file 1 [file mmc1.doc]

BJORL-D-23-00242_Supplementary Material

**Supplemental Table 1** Clinical-epidemiological variables for statistical analysis of patients with oral or oropharyngeal mucosal lesions attended at the INI-FIOCRUZ from 2005 to 2017.

| **Variable** | **Definition** | **Categories** |
| --- | --- | --- |
| Age | In years | In years |
| Gender | Biological | Male |
|  |  | Female |
| Skin color | Self-declared | White |
|  |  | Feoderm |
|  |  | Black |
| Education level | Self-declared | Illiterate up to last year of elementary school |
|  |  | Middle school |
|  |  | High school, associate degree, undergraduate degree, and graduate school |
| Origin | Self-declared | Rio de Janeiro city and metropolitan region |
|  |  | Rio de Janeiro state |
|  |  | Other states |
| Smoking | Data obtained according to the registry in the medical record; no information on the amount and frequency of use | Yes |
|  |  | No |
| Alcohol use | Data obtained according to the registry in the medical record; no information on the amount and frequency of intake | Yes |
|  |  | No |
| First mucosal sign/symptom presented by the patient | Self-declared | Local pain |
|  |  | Odynophagia |
|  |  | Bleeding |
|  |  | Dysphagia |
|  |  | Absence of local pain |
| Time of disease evolution | The time (in months) between the emergence of the oral and oropharyngeal mucosal lesion and diagnosis | In months |
| HIV co-infection | Serological confirmation | HIV-positive |
|  |  | HIV-negative |

**Supplemental Table 2** Age distribution of patients with oral or oropharyngeal mucosal lesions in each disease group attended at the INI-FIOCRUZ from 2005 to 2017.

|  | **Developmental disturbances (n = 3)** | | **Non-granulomatous infectious diseases (n = 220)** | | **Granulomatous infectious diseases (n = 155)** | | **Autoimmune diseases (n = 24)** | | **Benign neoplasms (n = 13)** | | **Malignant neoplasms (n = 103)** | | **NCOC diseases^a^ (n = 102)** | | **Total (n = 620)** | |
| --- | --- | --- | --- | --- | --- | --- | --- | --- | --- | --- | --- | --- | --- | --- | --- | --- |
|  | **n** | **%** | **n** | **%** | **n** | **%** | **n** | **%** | **n** | **%** | **n** | **%** | **n** | **%** | **n** | **%** |
| 0–9^b^ | 0 | 0 | 10 | 4.5 | 0 | 0. | 0 | 0 | 0 | 0 | 0 | 0 | 7 | 6.9 | 17 | 2.7 |
| 10–19 | 0 | 0 | 23 | 10.5 | 2 | 1.3 | 0 | 0 | 0 | 0 | 0 | 0 | 9 | 8.8 | 34 | 5.5 |
| 20–29 | 0 | 0 | 51 | 23.2 | 10 | 6.5 | 3 | 12.5 | 1 | 7.7 | 7 | 6.8 | 12 | 11.8 | 84 | 13.5 |
| 30–39 | 0 | 0 | 41 | 18.6 | 14 | 9 | 1 | 4.2 | 3 | 23.1 | 4 | 3.9 | 22 | 21.6 | 85 | 13.7 |
| 40–49 | 1 | 33.3 | 42 | 19.1 | 40 | 25.8 | 5 | 20.8 | 6 | 46.2 | 18 | 17.5 | 21 | 20.6 | 133 | 21.5 |
| 50–59 | 2 | 66.7 | 31 | 14.1 | 48 | 31 | 7 | 29.2 | 3 | 23.1 | 29 | 28.2 | 15 | 14.7 | 135 | 21.8 |
| 60–69 | 0 | 0 | 15 | 6.8 | 30 | 19.4 | 2 | 8.3 | 0 | 0 | 24 | 23.3 | 11 | 10.8 | 82 | 13.2 |
| 70–79 | 0 | 0 | 7 | 3.2 | 10 | 6.5 | 5 | 20.8 | 0 | 0 | 14 | 13.6 | 5 | 4.9 | 41 | 6.6 |
| ≥80 | 0 | 0 | 0 | 0 | 1 | 0.6 | 1 | 4.2 | 0 | 0 | 7 | 6.8 | 0 | 0 | 9 | 1.5 |

^a^ NCOC diseases - epithelial and soft tissue diseases not classified in other categories.

^b^ In years.

**Supplemental Table 3** Information on residence/labor activity in urban or rural areas of patients with oral or oropharyngeal mucosal lesions of granulomatous infectious disseases attended at the INI-FIOCRUZ from 2005 to 2017.

|  | **Leprosy** | | **Histoplasmosis** | | **Sporotrichosis** | | **Tuberculosis** | | **American tegumentary leishmaniasis** | | **Paracoccidioidomycosis** | | **Total (n=123)** | |
| --- | --- | --- | --- | --- | --- | --- | --- | --- | --- | --- | --- | --- | --- | --- |
|  | **n** | **%** | **n** | **%** | **n** | **%** | **n** | **%** | **n** | **%** | **n** | **%** | **n** | **%** |
| **Urban^1^** | ‒^a^ | ‒ | 2 | 100 | 2 | 100 | 6 | 100 | 36 | 70.6 | 40 | 64.5 | 86 | 69.9 |
| **Rural^2^** | ‒ | ‒ | ‒ | ‒ | ‒ | ‒ | ‒ | ‒ | 15 | 29.4 | 22 | 35.5 | 37 | 30.1 |

^1^ Labor activity or residing in urban areas.

^2^ Labor activity or residing in rural areas.

^a^ Information not available.

**Supplemental Table 4** First mucosal sign/symptom presented by the patients with oral or oropharyngeal mucosal lesions attended at the INI-FIOCRUZ from 2005 to 2017.

|  |  | **Developmental disturbances** | | **Non-granulomatous infectious diseases** | | **Granulomatous infectious diseases** | | **Autoimmune diseases** | | **Benign neoplasms** | | **Malignant neoplasms** | | **NCOC diseases^a^** | | **Total (n^1^=286)** | |
| --- | --- | --- | --- | --- | --- | --- | --- | --- | --- | --- | --- | --- | --- | --- | --- | --- | --- |
|  |  | **n** | **%** | **n** | **%** | **n** | **%** | **n** | **%** | **n** | **%** | **n** | **%** | **n** | **%** | **n** | **%** |
| **1^st^ Symptom^2,3^** | Local pain | 0 | 0 | 35 | 47.3 | 19 | 21 | 10 | 62.5 | 0 | 0 | 35 | 46.7 | 13 | 29.6 | 112 | 39.2 |
|  | Odynophagia | 0 | 0 | 35 | 47.3 | 24 | 26.3 | 2 | 12.5 | 1 | 25 | 23 | 30.7 | 8 | 18.8 | 93 | 32.5 |
|  | Bleeding | 0 | 0 | 0 | 0 | 3 | 3.3 | 0 | 0 | 0 | 0 | 2 | 2.7 | 0 | 0 | 5 | 1.8 |
|  | Dysphagia | 0 | 0 | 1 | 1.3 | 10 | 11 | 0 | 0 | 0 | 0 | 3 | 4 | 0 | 0 | 14 | 4.9 |
|  | No pain^4^ | 1 | 100 | 3 | 4 | 35 | 38.5 | 4 | 25 | 3 | 75 | 12 | 16 | 23 | 52.4 | 87 | 30.4 |

^a^ NCOC diseases - epithelial and soft tissue diseases not classified in other categories.

^1^ Number of patients with available information.

^2^ First mucosal sign/symptom presented by the patients.

^3^ May be more than 1 first symptom per patient.

^4^ Absence of local pain.

**Supplemental Table 5** HIV co-infection of patients with oral or oropharyngeal mucosal lesions attended at the INI-FIOCRUZ from 2005 to 2017.

|  | **Developmental disturbances** | | **Non-granulomatous infectious diseases** | | **Granulomatous infectious diseases** | | **Autoimmune Diseases** | | **Benign neoplasms** | | **Malignant neoplasms** | | **NCOC diseases^a^** | | **Total (n^1^=203)** | |
| --- | --- | --- | --- | --- | --- | --- | --- | --- | --- | --- | --- | --- | --- | --- | --- | --- |
|  | **n** | **%** | **n** | **%** | **n** | **%** | **n** | **%** | **n** | **%** | **n** | **%** | **n** | **%** | **n** | **%** |
| **HIV^2^ positive** | 2 | 66.7 | 38 | 74.5 | 17 | 24.3 | ‒ | ‒ | 7 | 87.5 | 13 | 43.3 | 36 | 97.3 | 113 | 55.7 |
| **HIV negative** | 1 | 33.3 | 13 | 25.5 | 53 | 75.7 | 4 | 100 | 1 | 12.5 | 17 | 56.7 | 1 | 2.7 | 90 | 44.3 |

^a^ NCOC diseases - epithelial and soft tissue diseases not classified in other categories.

^1^ Number of patients with available information.

^2^ HIV co-infection.

**Supplemental Figure 1** Distribution by age groups of patients with oral and/or oropharyngeal mucosal attended at the INI-FIOCRUZ from 2005 to 2017.
